# Ethnic and racialized disparities in the use of screening services for pap smears and mammograms in Canada

**DOI:** 10.1002/cam4.70021

**Published:** 2024-10-25

**Authors:** Bukola O. Salami, Cindy Z. Kalenga, Mary Olukotun, Andre M. N. Renzaho, Aloysius Nwabugo Maduforo, Jesus A. Serrano‐Lomelin, Modupe Tunde‐Byass, Regine U. King, Solina Richter, Tehseen Ladha, Ambikaipakan Senthilselvan, Paul Bailey, Maria B. Ospina

**Affiliations:** ^1^ Department of Community Health Sciences University of Calgary Calgary Alberta Canada; ^2^ Cumming School of Medicine University of Calgary Calgary Alberta Canada; ^3^ Faculty of Nursing University of Alberta Edmonton Alberta Canada; ^4^ Translational Health Research Institute Western Sydney University Campbelltown New South Wales Australia; ^5^ Department of Public Health Sciences Queens University Kingston Ontario Canada; ^6^ Department of Obstetrics and Gynecology University of Toronto Toronto Ontario Canada; ^7^ Faculty of Social Work University of Calgary Calgary Alberta Canada; ^8^ College of Nursing University of Saskatchewan Saskatoon Saskatchewan Canada; ^9^ Department of Pediatrics University of Alberta Edmonton Alberta Canada; ^10^ School of Public Health University of Alberta Edmonton Alberta Canada; ^11^ Black Health Alliance Toronto Ontario Canada

**Keywords:** Canadian healthcare, cancer screening, ethnic disparities, racialized communities, women's health

## Abstract

**Background:**

Breast and cervical cancers pose significant health challenges for women globally, emphasizing the critical importance of effective screening programs for early detection. In Canada, despite the implementation of accessible healthcare systems, ethnic and racialized disparities in cancer screening persist. This study aims to assess ethnic and racialized disparities in breast and cervical cancer screening in Canada.

**Methods:**

Using 2015–2019 data from the Canadian Community Health Survey, we analyzed women aged 18–70 in distinct ethnic and racial groups. The primary outcome was mammography or Papanicolaou test (pap smear). The secondary outcome was time since the last screening. We used weighted multivariable logistic regression to estimate the odds of having a pap smear or mammography across the ethnic and racialized groups, adjusted for relevant covariates. Results were reported as odds ratios (ORs) with 95% confidence intervals (CIs).

**Results:**

We included 14,628,067 women of which 72.5% were White, 8.4% Southeast Asian, 4.7% South Asian, 3.4% Indigenous, 2.7% Black, 2.0% West Asian, and 1.6% Latin American. In comparison with the White reference group, a higher odds ratio of not having a pap smear was estimated for the West Asian (5.63; CI 3.85, 8.23), South Asian (5.19; CI 3.79, 7.12), Southeast Asian (4.35; CI 3.46, 5.46), and Black groups (2.62; CI 1.82, 3.78). Disparities in mammography screening were found only for the Southeast Asian group with higher odds of not having screening (1.85; CI 1.15, 2.98) compared to the White reference group.

**Conclusion:**

This study reveals significant disparities in pap smear and mammography screenings affecting various ethnic groups, particularly in West Asia, South Asian, and Black communities. These findings underscore the urgent need for targeted interventions, policies, and healthcare strategies to address these gaps and ensure equitable access to essential breast and cervical cancer prevention across all ethnicity.

## Graphical abstract

This study investigates ethnic and racialized disparities in breast and cervical cancer screenings in Canada using population‐based data from 2015 to 2019. Findings reveal substantial screening gaps, particularly affecting West Asian, South Asian, and Black communities, emphasizing the urgent need for targeted interventions to ensure equitable access to essential preventive measures across all ethnicities.

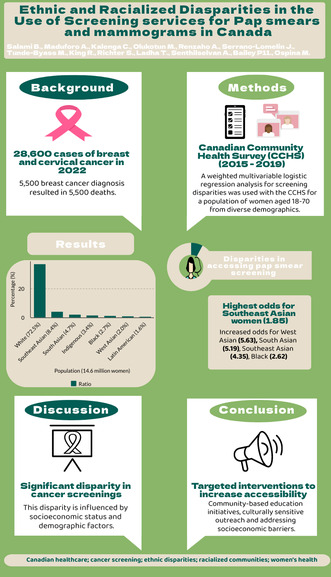

## 1 INTRODUCTION

Cancer stands as the world's second leading cause of death, claiming an estimated 9.6 million lives in 2018. Globally, approximately one out of every six deaths can be attributed to cancer.^1^ Breast, colorectal, lung, cervical, and thyroid cancer are the leading types of cancers observed among women.^1^ Cancer is the top cause of death in Canada, responsible for 28.2% of all fatalities.^2^ The most prevalent cancers are lung, breast, colorectal, and prostate, comprising 46% of new cases.^2^ Prostate, lung, breast, and colorectal cancer account for 20%, 13%, 25%, and 10% of new cases, respectively.^2^ In 2022, around 28,600 Canadian women are estimated to be diagnosed with breast cancer, leading to approximately 5500 deaths. This cancer represents 25% of new cancer cases and 14% of all cancer‐related deaths in Canadian women.^3^, ^4^ The statistics highlight a significant risk, with 1 in 8 women expected to develop breast cancer in their lifetime and 1 in 34 facing mortality from the disease.^4^ These figures emphasize the critical need for ongoing awareness, prevention, and treatment efforts to combat breast cancer's impact on women's health in Canada as well as underscore the critical importance of addressing cancer as a public health issue in Canada.^2^, ^3^, ^4^

Early detection and treatment significantly reduce cancer mortality rates.^1^, ^5^, ^6^ The Canadian Cancer Society recommends that women aged 40–74 undergo a mammogram every 2 years.^7^ The Canadian Cancer Society also recommends screening for cervical cancer with a pap smear for women aged 25–69 every 3 years, with screening to begin as early as 21 years if sexually active or in a high‐risk group.^8^ Due to the importance of early detection of precancerous conditions in the cervix and vagina with pap smear test, the Canadian Cancer Society previously recommended “a pap test done as part of a regular checkup or during a pelvic exam.”^9^ Although the current recommendation is every 3 years.^10^

The recommendations on the age to begin mammograms and the frequency of screenings differ across provinces in Canada.^11^ The breast cancer screening recommendations vary based on age and risk factors, with provincial screening programs differing across regions. For instance, in Ontario, the Ontario Breast Screening Program (OBSP) is a provincial initiative recommending regular mammograms for individuals aged 50–74 every 2 years. Notably, starting in fall 2024, those aged 40–49 can self‐refer for mammograms, signaling a move toward enhanced accessibility in screening. For individuals at confirmed high risk, like those aged 30–69 with pertinent family or medical histories, annual mammograms with MRI or ultrasound are advised through OBSP.^12^ These efforts reflect a structured approach to population‐based screening, alongside provisions for opportunistic screening among higher‐risk groups, aiming to bolster early detection and improve breast cancer management outcomes nationwide.

Racialized individuals, also known as visible minorities in Canada, are defined in the Canadian Employment Equity Act as persons, other than Aboriginal peoples, who are non‐Caucasian in race or non‐white in color.^13^, ^14^ This definition includes South Asian, Chinese, Black, Filipino, Arab, Latin American, Southeast Asian, West Asian, Korean, and Japanese populations.^13^ In 2021, 25% of Canada's population belonged to racialized groups, with South Asian, Chinese, and Black individuals accounting for 16.1%.^13^ This marks significant growth from the 13.4% recorded in 2001.^15^ Unfortunately, racialized Canadians, particularly Black individuals, face substantial health disparities, including high rates of chronic conditions and limited access to healthcare services.^16^, ^17^, ^18^, ^19^, ^20^, ^21^, ^22^, ^23^, ^24^, ^25^ Mental health illness and cardiovascular disease disparities are notable among racialized women.^16^, ^20^, ^22^, ^25^ These issues stem from systemic racism, socioeconomic challenges, and education and employment gaps.^23^, ^24^, ^25^

Evidence suggests that the intersection of race and other social determinants of health plays a significant role in the well‐being of Black and racialized Canadians; however, there is limited data regarding its impact on healthcare access. Furthermore, there is no published national study on the utilization of screening services for pap smears and mammograms among Black and racialized Canadians. Therefore, this study examines ethnic and racialized disparities in the use of screening services for pap smears and mammograms among women in Canada.

## 2 METHOD

### 2.1 Study design and data sources

We conducted a secondary cross‐sectional analysis of data from the Canadian Community Health Survey (CCHS)^26^ to estimate ethnic and racial disparities in breast and cervical cancer screening in Canada between 2015 to 2019. The study used microdata files from the CCHS that were accessed through the Statistics Canada Research Data Centres (RDC) located at the University of Alberta (Edmonton) and Queen's University (Kingston). The CCHS is a cross‐sectional, self‐reported survey conducted by Statistics Canada every year to gather health‐related data across Canada. It covers approximately 98% of the population aged 12 years and older from all provinces and territories, excluding persons living on reserves, full members of the Canadian forces, individuals living in long‐term institutions, and in some remote regions. Combined data from cycles 2015 to 2019 were used for the study as those years shared a common sampling methodology.

### 2.2 Study population

The study sample included respondents who self‐identified as women in the CCHS survey cycles from 2015 to 2019, aged between 18 and 70 years at the time of the survey, and who identified themselves as belonging to any of the following ethnic/racialized groups: Black, White, Indigenous (First Nations, Métis or Inuk (Inuit)), Southeast Asian (Chinese, Filipino, Vietnamese, Cambodian, Malaysian, Laotian, Thai, Korean, Japanese), Arab or West Asian (Arab, Iranian, Afghan), Latin American, South Asian (e.g., East Indian, Pakistani, Sri Lankan), and Other or not declared. The sample included household weights for each year, which are representative of the whole target population and are provided by Statistics Canada. We adjusted the weights to get the average population of women aged 18 years and above from 2015 to 2019.

### 2.3 Measures

The main study outcome was having a mammography or a Papanicolaou test (pap smear). The secondary outcome was time (in years) of the last screening. Tables S1 and S2 in Appendix S1 present detailed information for each variable. Our primary independent variable was self‐perceived race and ethnicity. We used age, education, income, and landed immigrant status as covariates. Age was aggregated into four categories: 18–30, 31–49, 50–64, and ≥65 years. Education attainment included three categories: less than secondary school graduation, secondary school graduation, and certificate diploma or university degree. The household income ratio is the adjusted ratio of the total household income of the respondent to the low‐income cut‐off corresponding to their household and community size. It is calculated by Statistics Canada and presented in deciles from the smallest (most economically disadvantaged) to the largest (less economically disadvantaged) ratio.^27^ We aggregated the deciles into quintiles to increase the sample size per category. Landed immigrant status in Canada has five categories: Yes, No, Valid Skip (for respondents born in Canada), Don't know, Refuse to answer, and Not stated. The categories presented in this manuscript for all variables were based on reaching enough sample size to comply with Statistics Canada vetting rules and to improve statistical estimates.

### 2.4 Statistical analysis

We first described the ethnic and racialized group composition for the 2015–2019 combined sample and the demographic characteristics for women 18 years or older using cross‐classification tables. We reported weighted percentages (%), 95% confidence intervals (CIs), and the average population representative from the combined sample. For pap smear and mammography screening, we reported weighted percentages (%), 95% CI, and the average population representative from two subsamples: women 21–70 years old for the analysis of pap smear screening and women 50–74 years old for mammography screening. We conducted a weighted multivariable logistic regression to calculate adjusted odds ratios (aOR) of having a pap smear or a mammography across the ethnic and racialized groups relative to the White group as the reference category and adjusting for the previously described covariates. Results deemed too unreliable for publication are marked with “***” in tables and figures, indicating that the coefficient of variation (CV) significantly exceeded 16%. This might be caused by CCHS sample design effect and/or small sample that do not meet Statistics Canada's confidentiality vetting requirements and were omitted from tables. All statistical analyses were conducted using Stata Statistical Software: Release 13 (College Station, TX: StataCorp LP).

## 3 RESULTS

**FIGURE 1:**
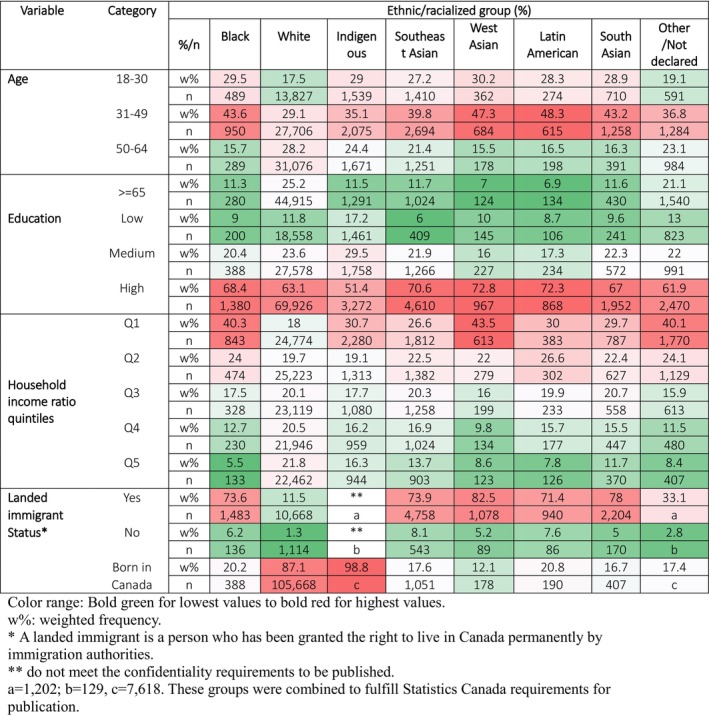
Heat map of demographic characteristics by cultural group. Women 18 years old and above. 2015–2019 combined sample. Observations = 142,244; Weighted population size = 14,628,067.

**FIGURE 2:**
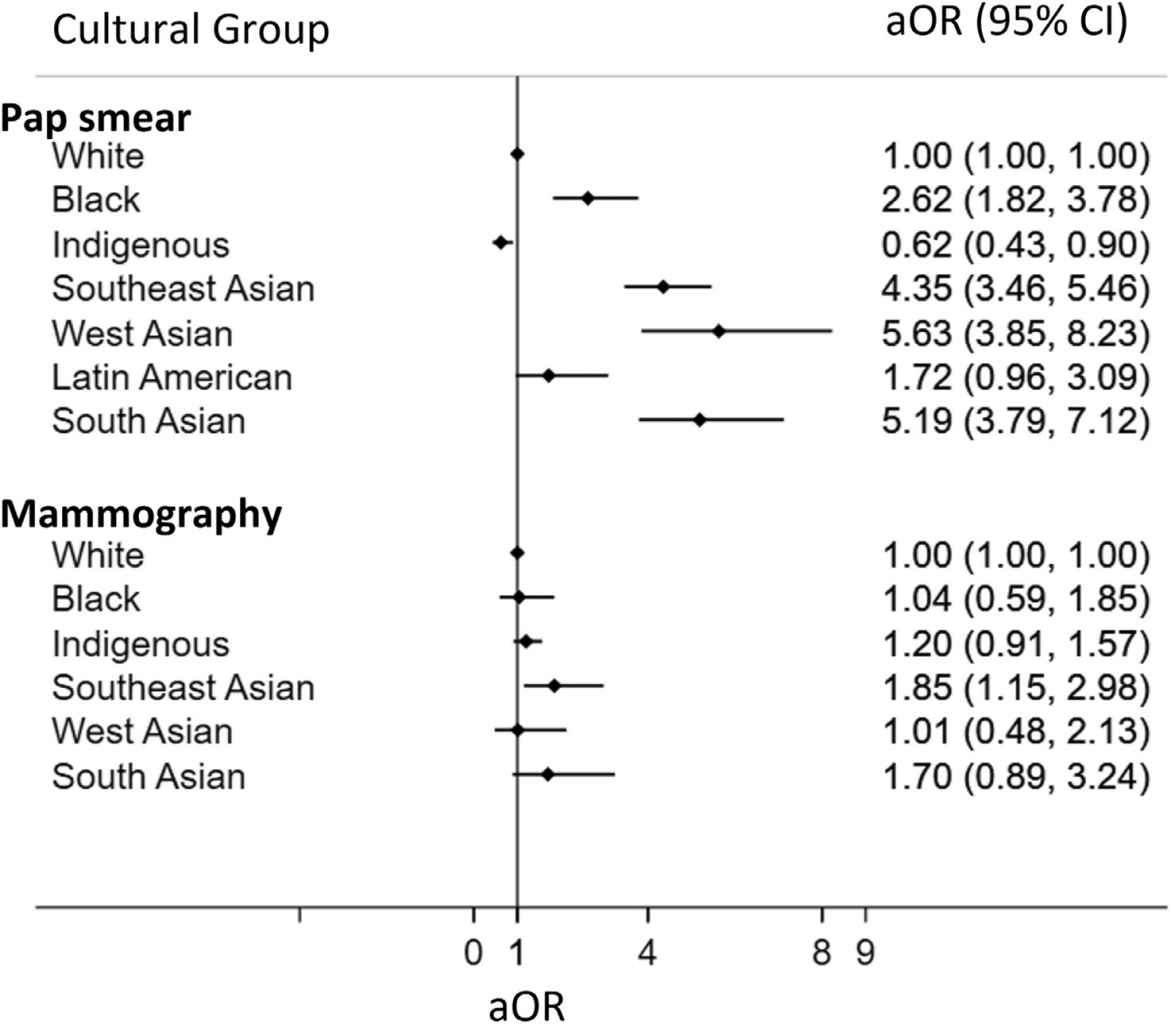
Adjusted odds ratio (aOR) of not having pap smear in lifetime or not have been screened for breast cancer in life time. Odds ratio adjusting for age, education level, and household income levels.

The study population included a weighted population of 14,628,067 women aged 18 years and older from 2015 to 2019. The results reveal a varied demographic composition, with the following percentages attributed to different ethnic and racial groups: White (72.5%), Southeast Asian (8.4%), South Asian (4.7%), Indigenous (3.4%), Black (2.7%), West Asian (2.0%), and Latin American (1.6%) (Appendix Figure S1). Additionally, the data indicates that 4.8% of the sample did not identify with any specific ethnic or racialized group, highlighting the diversity and complexity of the Canadian population. Figure 2 shows that the age composition of White women was scattered across all age categories; among racial and ethnic groups, the largest percentage of women ≥65 years of age (25.2%) and the lowest percentage of women aged 18–30 (17.5%) was among White women. The proportion of women aged 18–30 within each racial and ethnic group was otherwise comparable, ranging from 27.2% (Southeast Asian) to 30.2% (West Asian). The age group of 31–49 years was the most frequent for all Ethnic/Racialized groups: White (29.1%), Latin American (48.3%), West Asian (47.3%), Black (43.6%), South Asian (43.2%), Southeast Asian (39.8%), and Indigenous (35.1%). Educational attainment was high among all groups (Figure 1), as the data indicates that most of the women had a certificate diploma or university degree: West Asian (72.8%), Latin American (72.3%), Southeast Asian (70.6%), South Asian (67%), Black (68.4%), White (63.1%), and Indigenous (51.4%). Household income ratio (Figure 1) distribution across quintiles was uniform for White women, with percentages ranging from 18% to 21.8%. On average, other ethnic/racialized groups had lower household income ratio quintiles: West Asian (43.5%), Black (40.3%), Indigenous (30.7%), Latin American (30%), and South Asian (29.7%), and Southeast Asian (26.6%). Landed immigrant status data (Figure 1) indicates that women from the Indigenous and White groups were mainly born in Canada (98.8% and 87.1%, respectively). Most of women from the other ethnic/racialized groups had a landed immigrant status: West Asian (82.5%), South Asian (78%), Black (73.6%), Southeast Asian (73.9%), and Latin American (71.4%).

### 3.1 Cervical cancer screening

The result from a study focusing on Pap smear test utilization among women aged 21–70 across diverse cultural groups, encompassing a weighted population of 3,119,520 women. Disparities in pap smear screening were observed across ethnic and racial groups (Figure 2 and Appendix Table S2). Among the White and Indigenous groups, 7% and 6% of women, respectively, had never undergone a pap smear in their lifetime. However, notably higher percentages were found among other ethnic groups: West Asian (30.3%), South Asian (27.4%), Southeast Asian (22.7%), and Black (18.1%) (Appendix Table S2). Of those who had been screened, most women from all groups had received a pap smear within the last 2 years. However, the White and Indigenous groups exhibited higher percentages of women who had not had a pap smear within this timeframe, at 32.9% and 28.4%, respectively. Conversely, ethnic and racial minority groups displayed more consistent rates of screening within 2 years: Black (22%), Southeast Asia (24.8%), West Asian (19.2%), South Asian (22.7%), Other/not declared (26.7%) (Appendix Table S2).

Belonging to certain ethnic or racialized group was associated with significantly higher odds of never having received a pap test West Asia (aOR 5.63; 95% CI 3.85, 8.23), South Asia (aOR 5.19; 95% CI 3.79, 7.12), Southeast Asia (aOR 4.35; 95% CI 3.46, 5.46), Black (aOR 2.62; 95% CI 1.82, 3.78), Latin America (aOR 1.72; 95% CI 0.96, 3.09) (Figure 2 and Table 1). Conversely, being of Indigenous background was associated with lower odds of never having received a pap test (aOR 0.62; 95% CI 0.43, 0.90).

**TABLE 1:** Adjusted odds (multivariable model) of **Not having a Pap smear test** during lifetime among cultural groups. Women 21–70 years (2015–2019).

Variable	Category	Adjusted OR	95% CI	*p*‐Value
Cultural group				
	White (reference)	1		
	**Black**	**2.62**	**(1.82, 3.78)**	**<0.01**
	**Indigenous**	**0.62**	**(0.43, 0.90)**	**0.012**
	**Southeast Asian**	**4.35**	**(3.46, 5.46)**	**<0.01**
	**West Asian**	**5.63**	**(3.85, 8.23)**	**<0.01**
	Latin American	1.72	(0.96, 3.09)	0.068
	**South Asian**	**5.19**	**(3.79, 7.12)**	**<0.01**
	**Other/Not declared**	**2.06**	**(1.31, 3.23)**	**<0.01**
Age				
	18–30 (reference)	1		
	**31–49**	**0.24**	**(0.20, 0.28)**	**<0.01**
	**50–64**	**0.23**	**(0.19, 0.28)**	**<0.01**
	**≥65**	**0.33**	**(0.25, 0.42)**	**<0.01**
Education				
	Diploma or university degree (reference)	1		
	**Less than secondary**	**2.46**	**(1.91, 3.17)**	**<0.01**
	**Secondary**	**1.41**	**(1.16, 1.71)**	**<0.01**
Household income ratio				
	Q5 (most advantaged: reference)	1		
	Q4	1.09	(0.83, 1.43)	≫0.05
	Q3	1.12	(0.87, 1.46)	≫0.05
	**Q2**	**1.32**	**(1.01, 1.73)**	**0.042**
	**Q1 (most disadvantaged)**	**1.78**	**(1.38, 2.31)**	**<0.01**

Bold indicates *p* < 0.05.

Furthermore, the multivariable model revealed that lower education and lower household income increased the odds of not having a pap test in a lifetime (Table 1). Women with less than secondary education (aOR = 2.46; 95% CI 1.91, 3.17) or secondary education (aOR = 1.41; 95% CI 1.16, 1.71) were more likely to have never had a pap smear compared to those with certificate diplomas or university degrees. Similarly, higher odds of never having had a pap smear were observed for women with lower household income ratios (aOR = 1.78; 95% CI 1.38, 2.31 for Q1; aOR = 1.32; 95% CI 1.01, 1.73 for Q2) compared to women with the highest household income ratio (Q5) (Table 1). Older age was associated with lower odds of never being screened in compared to the younger age group.

### 3.2 Breast cancer screening

The data on mammography screening among women aged 50 to 74 from various cultural groups, with a weighted population size of 2,733,152. Significant disparities in mammography screening were also noted among ethnic and racial groups (Figure 2, Table 2, and Appendix Table S4). Compared to the White group, higher percentages of women from Southeast Asia (19.5%), South Asia (19.2%), West Asia (13.1%), Indigenous (11.6%), and Black (10.8%) groups had never undergone breast cancer screening (Appendix Table S4). Most women had received a mammogram within the last 2 years, but the Southeast Asian and Indigenous groups had higher percentages of women who hadn't, at 29.6% and 27.5%, respectively. Other groups had more similar rates of screening within the last 2 years: White (23.3%), Black (21.5%), South Asian (20.8%), and West Asian (13.4%) (Appendix Table S4).

**TABLE 2:** Adjusted odds (multivariable model) of **Not screening for breast cancer** among cultural groups. Women 50–74 years (2015–2019).

Variable	Category	Adjusted OR	95% CI	*p*‐Value
Cultural group				
	White (reference)	1		
	Black	1.04	(0.59, 1.85)	≫0.05
	Indigenous	1.20	(0.91, 1.57)	≫0.05
	**Southeast Asian**	**1.85**	**(1.15, 2.98)**	**0.012**
	West Asian	1.01	(0.48, 2.13)	≫0.05
	South Asian	1.70	(0.89, 3.24)	≫0.05
	Other/Not declared	1.10	(0.68, 1.79)	≫0.05
Age				
	50–64 (reference)	1		
	**≥65**	**0.45**	**(0.39, 0.53)**	**<0.01**
Education				
	Diploma or University degree (reference)	1		
	Less than secondary	**1.51**	**(1.22, 1.88)**	**<0.01**
	Secondary	1.11	(0.93, 1.31)	≫0.05
Income				
	Q5 (most advantaged: reference)	1		
	Q4	1.20	(0.91, 1.59)	≫0.05
	Q3	1.12	(0.84, 1.49)	≫0.05
	Q2	**1.37**	**(1.04, 1.81)**	**0.025**
	Q1 (most disadvantaged)	**1.82**	**(1.37, 2.41)**	**<0.01**

Bold indicates *p* < 0.05.

Compared to the White reference group, the Southeast Asian group had significantly higher odds (aOR = 1.85, CI 1.15, 2.98) of never being screened for breast cancer, after adjusting for education, income, and immigration status (Figure 2).

In the multivariable model, lower education and lower household income increased the odds of never being screened for breast cancer. Women with less than a secondary education level (aOR = 1.51; 95% CI 1.22, 1.88) and those in economically deprived quintiles (Q1 and Q2) (aOR = 1.82; 95% CI 1.37, 2.41 and aOR = 1.37; 95% CI 1.04, 1.81, respectively) were more likely not to have had a mammogram in their lifetime compared to the White‐origin group (Table 2).

## 4 DISCUSSION

This cross‐sectional analysis of CCHS data evaluated ethnic and racial disparities in cervical and breast cancer screening in Canada. The study uncovered notable disparities in cervical cancer screening rates, with odds of not having a pap smear in lifetime increased among the West Asian, South Asian, Southeast Asian, and Black ethnic groups. Additionally, disparities in mammography screening were present only in the Southeast Asian group. Lastly, an association was observed between lower education status and low household income with heightened disparities in both cervical and breast cancer screening, showing the association of socioeconomic factors in healthcare access. These findings highlight how racialized socio‐economic disadvantage can contribute negatively to use of health services. Racialized disparities in cancer screening can lead to racialized inequities in health outcomes. Indeed, data from Statistics Canada indicate that Black Canadians experience higher mortality rate from cancer than White Canadian, which may be as a result of disparities in cancer screening leading to delayed diagnosis.^28^

Racial disparities in cervical cancer incidence and mortality are large, especially among Black women, who are 30% more likely to develop and 60% more likely to die from cervical cancer than their White counterparts.^29^ Timely cervical cancer screening through pap smears is a powerful though underutilized tool for prevention and early detection. Numerous studies consistently underscore the existence of disparities in cervical cancer screening rates among ethnic and racial minorities.^30^, ^31^, ^32^, ^33^ Our study reveals that certain groups, specifically Black and Asian women, are less likely to undergo regular Pap smears compared to their White counterparts. A study of 984 women from 2009 to 2011,^33^ the authors observed high disparities in pap smear utilization, particularly pronounced among specific ethnic groups, underscoring the urgent need for targeted interventions to enhance cervical cancer screening accessibility and uptake. The substantial differences in pap smear rates between ethnic and racialized groups highlight a concerning pattern. These disparities indicate a potential barrier to preventive healthcare services within these communities, emphasizing the necessity for culturally sensitive outreach programs and community‐based education initiatives.

While overall mammography screening rates were relatively high (76.4%) among women aged 50 to 74 years, surpassing Canada's target participation rate of ≥70%,^34^ significant disparities were evident for the Southeast Asian group. Nearly one‐fifth (19.5%) of Southeast Asian women in this age group had not been screened for breast cancer in their lifetime, a statistic significantly higher than the general population. After adjusting for education, income, and immigration status, the Southeast Asian group exhibited higher odds of not being screened for breast cancer in their lifetime. Irrespective of the demographic, common obstacles exist that impede effective engagement with the healthcare system, such as difficulty in navigating healthcare processes (limited knowledge, absence of provider guidance, and low health literacy).^35^ In countries without universal health care, financial impediments, such as out‐of‐pocket expenses and lack of insurance coverage, compound these barriers. However, even where universal healthcare is provided, still logistical considerations, including conflicting priorities, absence of childcare, and scheduling complexities, collectively present additional hurdles for under screened groups in the cervical cancer screening.^35^ Lower trust in providers, in conjunction with a lack of provider recommendation, can often contribute to low cancer screening rates among Hispanic, non‐Hispanic Black, and non‐Hispanic White women in other cancer contexts.^36^, ^37^ Black women, in particular, cite trust in their provider and in the healthcare system as specific factors that contribute to cervical cancer screening behaviors.^38^, ^39^

The multivariable model reveals that low education and low household income increase the odds of not having a Pap smear in a lifetime, suggesting a complex interplay between socioeconomic factors and cervical cancer screening. Women with lower educational attainment and household income ratios demonstrate heightened vulnerability to underutilizing Pap smear services. These findings underscore the importance of addressing socioeconomic determinants to ensure equitable access to cervical cancer screening, advocating for targeted educational campaigns, and support systems tailored to economically disadvantaged populations. Similar to cervical cancer screening, low education, and low household income were identified as risk factors for not undergoing mammography screening. Women with less than a secondary education level are particularly affected. This reinforces the crucial role of socioeconomic factors in shaping healthcare utilization patterns, urging the development of comprehensive strategies to address barriers related to education and income.

Early cancer detection and treatment is one of the pathways for increasing cancer survival among underserved groups such as the Black population.^27^ In particular, targeted interventions which consider “provider‐, facility‐, and system‐level solutions may be most effective for reaching underscreened individuals,” such as those belonging to visible minority groups.^33^ Per our findings, educational attainment and income are two related socioeconomic factors with significant implications for access to preventative health services in part due to difficulty navigating the healthcare system or other logistical barriers to access. Considering that a majority of the non‐Indigenous and non‐White sample were first‐generation immigrants, it's also important to note the relationship between being foreign‐born, having lower educational level, and lower income which independently and jointly impact access to preventative health services such as cancer screening.^5^ For these populations, outreach programs may be effective at increasing uptake of screening, for instance, through community‐based events where screening is promoted or provided.^33^ Additionally, patients with lower levels of education are more likely to seek multiple sources of information to guide their health decisions^27^; multifactorial culturally sensitive strategies including campaigns and lay health educators may also more broadly address information needs within the community. We have presented findings from a national dataset on cervical and breast cancer screening. While our results are unique, they must be interpreted in light of the study's limitations. We analyzed data from the CCHS, which collects information solely from Indigenous people living off reserve. Previous research has indicated significant health disparities among Indigenous populations in Canada.^40^ The exclusion of Indigenous populations from our sample has the potential to limit generalizability of the results to this population. Additionally, the survey includes only Canadian citizens and permanent residents. Temporary foreign workers, refugee claimants, landed immigrants and those not yet permanent residents in Canada, were excluded from our sample, and their health may face additional barriers based on immigration policies. Furthermore, pap smears and mammograms are recommended by family doctors; therefore, access to appropriate primary care may pose as a barrier. Sample size limitations and vetting rules further constrained our ability to conduct more complex analyses. However, it is crucial to note that self‐reported cervical cancer screening has been shown to not only overestimate screening use compared to matched health record data but also produce a differential misclassification by race, leading to underestimates of disparities.^38^, ^39^

In conclusion, this study reveals alarming disparities in cervical and breast cancer screening rates among various ethnic and racialized groups in Canada. The intersectionality of these disparities with socioeconomic factors emphasizes the need for targeted interventions that consider both cultural nuances and economic circumstances. While our findings indicate racialized disparities in breast and cervical cancer screening at the intersection of income and education, the underlying reason for these disparities is not clear. We recommend further mixed methods research with specific ethnic groups to support the development of culturally responsive health promotion and screening practices for cancer. In addition to culturally sensitive measures to address disparities, our findings point to the need for targeted approaches for low income and low education communities. These approaches must attend to the social determinants of health that may constrain racialized communities screening practices. Implementing community‐specific outreach programs, culturally competent healthcare initiatives, and addressing socioeconomic determinants are pivotal steps toward achieving health equity in cancer screening across diverse populations. Policymakers, healthcare professionals, and community leaders must collaborate to implement these strategies and dismantle the barriers that perpetuate disparities in preventive healthcare access. Additionally, further research is warranted to explore the root causes of these disparities and evaluate the effectiveness of tailored interventions in mitigating them.

## AUTHOR CONTRIBUTIONS

**Bukola O. Salami:** Conceptualization (lead); data curation (lead); formal analysis (lead); funding acquisition (lead); investigation (lead); methodology (lead); project administration (lead); resources (lead); software (lead); supervision (lead); validation (lead); visualization (lead); writing – original draft (lead); writing – review and editing (lead). **Cindy Z. Kalenga:** Data curation (equal); validation (equal); writing – original draft (equal); writing – review and editing (equal). **Mary Olukotun:** Methodology (equal); project administration (equal); validation (equal); writing – original draft (equal); writing – review and editing (equal). **Andre M. N. Renzaho:** Conceptualization (equal); data curation (equal); formal analysis (equal); funding acquisition (equal); methodology (equal); supervision (equal); validation (equal); visualization (equal); writing – review and editing (equal). **Aloysius Nwabugo Maduforo:** Data curation (equal); validation (equal); visualization (equal); writing – original draft (equal); writing – review and editing (equal). **Jesus A. Serrano‐Lomelin:** Data curation (equal); formal analysis (equal); methodology (equal); validation (equal); visualization (equal); writing – review and editing (equal). **Modupe Tunde‐Byass:** Conceptualization (equal); funding acquisition (equal); methodology (equal); validation (equal); writing – review and editing (equal). **Regine U. King:** Conceptualization (equal); funding acquisition (equal); methodology (equal); validation (equal); writing – review and editing (equal). **Solina Richter:** Conceptualization (equal); funding acquisition (equal); methodology (equal); validation (equal); writing – review and editing (equal). **Tehseen Ladha:** Conceptualization (equal); data curation (equal); formal analysis (equal); funding acquisition (equal); methodology (equal); validation (equal); visualization (equal); writing – review and editing (equal). **Ambikaipakan Senthilselvan:** Conceptualization (equal); formal analysis (equal); funding acquisition (equal); methodology (equal); validation (equal); visualization (equal); writing – review and editing (equal). **Paul Bailey:** Conceptualization (equal); funding acquisition (equal); methodology (equal); validation (equal); writing – review and editing (equal). **Maria B. Ospina:** Conceptualization (equal); data curation (equal); formal analysis (equal); funding acquisition (equal); methodology (equal); supervision (equal); validation (equal); writing – review and editing (equal).

## FUNDING INFORMATION

Women and Children's Health Research Institute.

## CONFLICT OF INTEREST STATEMENT

The authors declare no conflict of interest.

## INSTITUTIONAL REVIEW BOARD STATEMENT

The study was conducted in accordance with the Declaration of Helsinki and approved by the Institutional Review Board (or Ethics Committee) of the University of Alberta (protocol code Pro00115790).

## INFORMED CONSENT STATEMENT

Informed consent was obtained from all subjects involved in the study by Statistics Canada personnel.

## Supporting information


Appendix S1.

## Data Availability

Data for this study is available by contacting Statistics Canada. This article is based on a secondary analysis of the Canadian Health Measures Survey. Access to data from the Canadian Health Measures Survey is available through Statistics Canada.
