# Supplementary material for: Ethnic and racialized disparities in the use of screening services for pap smears and mammograms in Canada
Source: Cancer Med. 2024 Oct 25;13(20):e70021. doi: 10.1002/cam4.70021 (PMC11503031; doi:10.1002/cam4.70021)
Supplement: Supplementary file 1 — Appendix S1. [file CAM4-13-e70021-s001.docx]

**Appendix S1**


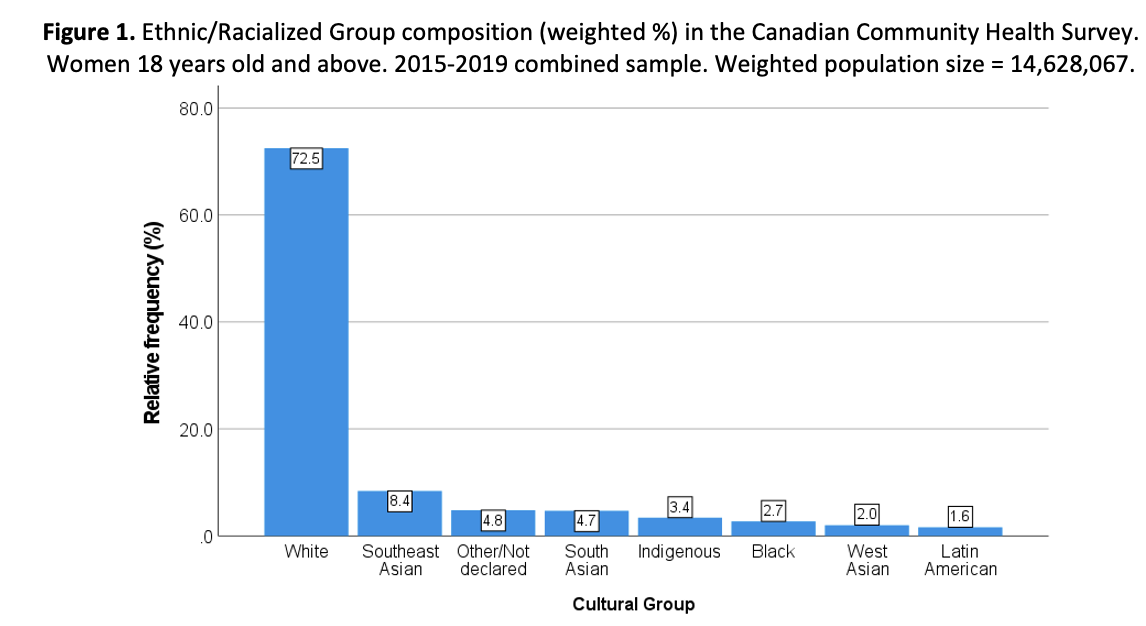


**Table S1.** Methodological characteristics for (A) pap smear and (B) mammography measures.


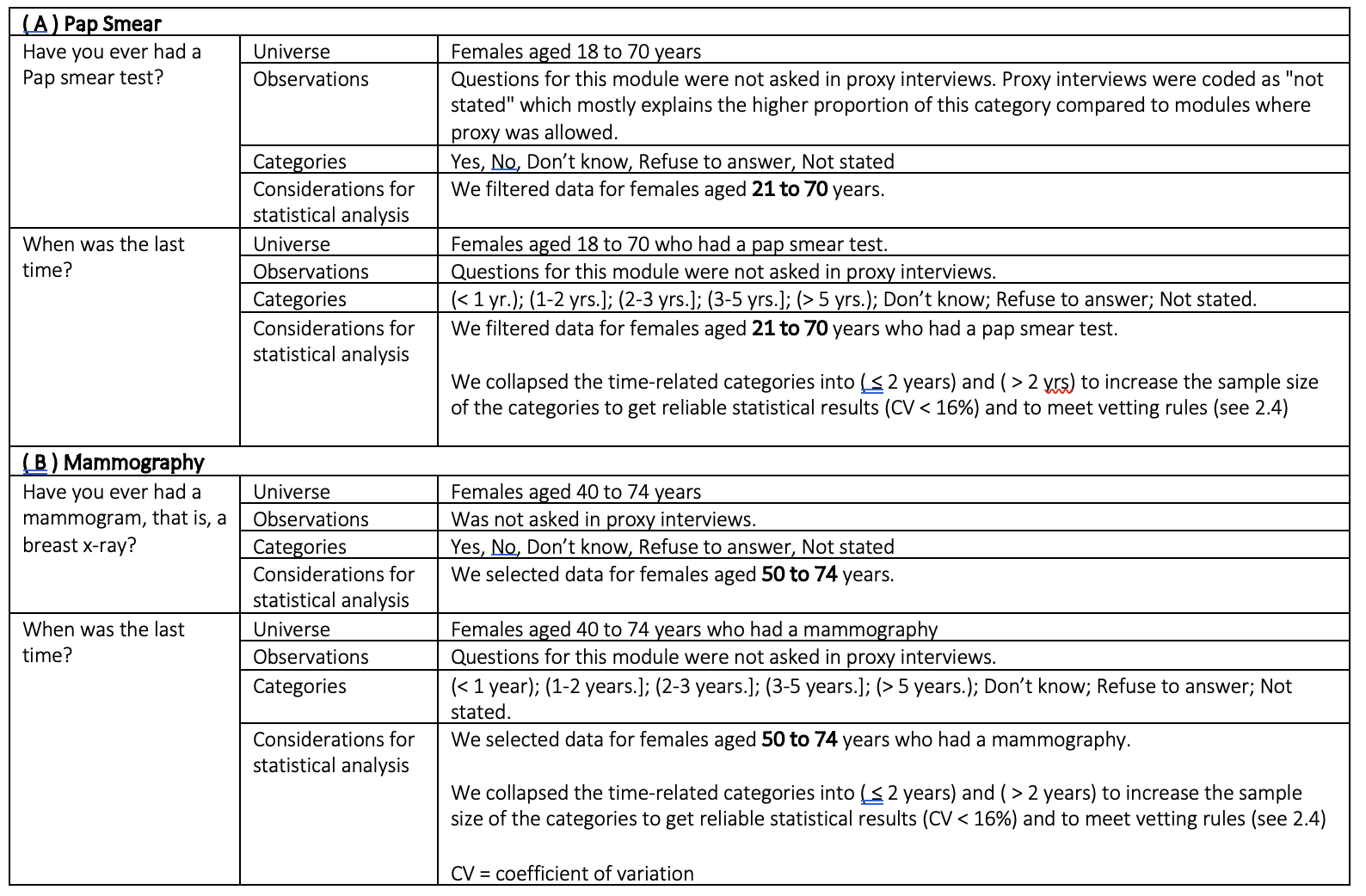


**Table S2**. Pap Smear Test. Women 21 to 70 years old. Observations = 30,040, Weighted population size = 3,119,520. Weighted frequencies expressed as percentages (%).

| **Question** | **Answer** | **%/CI/n** | **Black** | **White** | **Indigenou** | **Southeast Asian** | **West Asian** | **Latin American** | **South Asian** | **Other/Not declared** | **Total** |
| --- | --- | --- | --- | --- | --- | --- | --- | --- | --- | --- | --- |
| **Had a** | Yes | w% | 80.6 | 91.6 | 91.9 | 72.2 | 65.3 | 84.5 | 65.9 | 72.5 | 87.1 |
| **pap**  **smear** |  | 95%CI | 74.8,  85.3 | 91.0,  92.2 | 89.2,  93.9 | 68.4,  75.7 | 57.6,  72.2 | 76.8,  89.9 | 59.5,  71.7 | 65.4,  78.6 | 86.3,  87.8 |
| **test?** |  | n | 396 | 22,555 | 1,579 | 1,137 | 492* | | 503 | 576 | 27,238 |
|  | No | w% | 18.1 | 7.0 | 6.0 | 22.7 | 30.3 | ** | 27.4 | ** | 10.4 |
|  |  | 95%CI | 13.4,  23.8 | 6.4,  7.6 | 4.3,  8.4 | 19.5,  26.1 | 23.7,  37.8 |  | 22.0,  33.6 |  | 9.7,  11.1 |
|  |  | n | 74 | 1,447 | 83 | 361 | 129* | | 164 | 72 | 2,330 |
| **When** | ≤2yrs | w% | 77.0 | 66.4 | 71.1 | 73.4 | 79.3 | 72.7 | 74.1 | 72.1 | 68.1 |
| **was the**  **last** |  | 95%CI | 70.0,  82.7 | 65.4,  67.4 | 67.7,  74.3 | 68.9,  77.5 | 71.5,  85.4 | 53.1,  86.3 | 67.3,  79.9 | 66.3,  77.3 | 67.2,  69.0 |
| **time?** |  | n | 297 | 14,174 | 1,078 | 824 | 389* | | 364 | 374 | 17,500 |
|  | >2yrs | w% | 22.0 | 32.9 | 28.4 | 24.8 | 19.2 | ** | 22.7 | 26.7 | 30.8 |
|  |  | 95%CI | 16.3,  28.9 | 31.9,  33.9 | 25.2,  31.8 | 20.9,  29.2 | 13.4,  26.9 |  | 17.4,  28.9 | 21.6,  32.6 | 29.9,  31.7 |
|  |  | n | 93 | 8,191 | 491 | 300 | 98* | | 129 | 188 | 9,490 |

**Notes**: Had a pap smear test lifetime? Categories Yes and No included 97.5% of the study sub-population. The remained 2.5% correspond to the categories Don’t know, Refusal or Not stated.

When was the last time? Categories ≤ 2yrs and > 2yrs included 98.9% of the sub-population who had a pap smear test (87.1%). The remained 1.1% correspond to the categories Don’t know, Refusal or Not stated.

*These groups were combined to fulfill Statistics Canada requirements for publication.

** = do not meet the confidentiality requirements to be published.

w%: weighted frequency.

**Table S3**. Demographic characteristics of women 18 years old and above. 2015-2019 combined sample.

Weighted population size = 14,628,067. Weighted frequencies expressed as percentages (%) with 95% CI in squared parentheses.

|  |  | **Ethnic/Racialized groups** | | | | | | | |  |  |
| --- | --- | --- | --- | --- | --- | --- | --- | --- | --- | --- | --- |
| Variable |  | Black  % | White  % | Indigenous  % | Southeast  Asian % | West Asian  % | Latin American % | South Asian % | Other/Not declared % | Total  % | Cum  % |
| **Ethnic/** |  | 2.7  [2.5, 2.9] | 72.5  [72, 73] | 3.4  [3.2, 3.5] | 8.4  [8.1, 8.7] | 2.0  [1.9, 2.2] | 1.6  [1.4, 1.7] | 4.7  [4.4, 5.0] | 4.8  [4.6, 5.1] | 100 | 100 |
| **Age** | 18-30 | 29.5 | 17.5 | 29.0 | 27.2 | 30.2 | 28.3 | 28.9 | 19.1 | 20.1 | 20.1 |
|  |  | [26.3, 32.9] | [17.1, 17.9] | [27.1, 31.0] | [25.4, 29.0] | [26.6, 34.1] | [23.3, 33.8] | [26.3, 31.8] | [17.0, 21.3] | [19.7, 20.5] |  |
|  | 31-49 | 43.6 | 29.1 | 35.1 | 39.8 | 47.3 | 48.3 | 43.2 | 36.8 | 32.3 | 52.4 |
|  |  | [40.2, 47.1] | [28.7, 29.6] | [33.1, 37.1] | [37.8, 41.7] | [43.3, 51.3] | [43.4, 53.3] | [40.3, 46.1] | [34.2, 39.4] | [31.9, 32.8] |  |
|  | 50-64 | 15.7 | 28.2 | 24.4 | 21.4 | 15.5 | 16.5 | 16.3 | 23.1 | 25.9 | 78.3 |
|  |  | [13.2, 18.4] | [27.7, 28.6] | [22.6, 26.2] | [19.7, 23.3] | [12.6, 19.0] | [13.4, 20.2] | [14.0, 18.9] | [21.0, 25.4] | [25.5, 26.3] |  |
|  | >=65 | 11.3 | 25.2 | 11.5 | 11.7 | 7.0 | 6.9 | 11.6 | 21.1 | 21.7 | 100 |
|  |  | [9.4, 13.4] | [24.8, 25.6] | [10.4, 12.8] | [10.6, 12.8] | [5.4, 9.1] | [5.2, 9.0] | [10.0, 13.3] | [19.3, 23.0] | [21.4, 22.1] |  |
| **Education** | Low | 9.0 | 11.8 | 17.2 | 6.0 | 10.0 | 8.7 | 9.6 | 13.0 | 11.3 | 11.3 |
|  |  | [7.2, 11.1] | [11.5, 12.1] | [15.8, 18.6] | [5.2, 7.0] | [8.0, 12.5] | [6.3, 11.8] | [7.9, 11.6] | [11.5, 14.8] | [11.0, 11.6] |  |
|  | Medium | 20.4 | 23.6 | 29.5 | 21.9 | 16.0 | 17.3 | 22.3 | 22.0 | 23.1 | 34.4 |
|  |  | [17.7, 23.3] | [23.1, 24.0] | [27.6, 31.4] | [20.2, 23.6] | [13.3, 19.1] | [14.3, 20.9] | [20.0, 24.8] | [20.1, 24.1] | [22.8, 23.5] |  |
|  | High | 68.4 | 63.1 | 51.4 | 70.6 | 72.8 | 72.3 | 67 | 61.9 | 64.0 | 98.4 |
|  |  | [65.1, 71.6] | [62.7, 63.6] | [49.3, 53.5] | [68.7, 72.4] | [69.2, 76.2] | [68.1, 76.3] | [64.1, 69.8] | [59.4, 64.3] | [63.5, 64.4] |  |
| **Household** | Q1 | 40.3 | 18.0 | 30.7 | 26.6 | 43.5 | 30.0 | 29.7 | 40.1 | 22.1 | 22.1 |
| **Income** |  | [37.0, 43.8] | [17.6, 18.3] | [28.9, 32.6] | [24.9, 28.4] | [39.6, 47.5] | [25.9, 34.4] | [27.1, 32.5] | [37.6, 42.8] | [21.7, 22.4] |  |
| **ratio** | Q2 | 24.0 | 19.7 | 19.1 | 22.5 | 22.0 | 26.6 | 22.4 | 24.1 | 20.5 | 42.5 |
| **quintiles** |  | [21.1, 27.1] | [19.3, 20.0] | [17.6, 20.8] | [20.9, 24.2] | [18.8, 25.5] | [22.7, 31.0] | [20.1, 25.0] | [21.9, 26.3] | [20.1, 20.9] |  |
|  | Q3 | 17.5 | 20.1 | 17.7 | 20.3 | 16.0 | 19.9 | 20.7 | 15.9 | 19.7 | 62.3 |
|  |  | [14.9, 20.5] | [19.7, 20.5] | [16.1, 19.4] | [18.7, 22.0] | [13.1, 19.5] | [16.2, 24.1] | [18.3, 23.4] | [14.0, 18.0] | [19.3, 20.1] |  |
|  | Q4 | 12.7 | 20.5 | 16.2 | 16.9 | 9.8 | 15.7 | 15.5 | 11.5 | 18.9 | 81.2 |
|  |  | [10.6, 15.1] | [20.1, 20.9] | [14.7, 17.8] | [15.4, 18.5] | [7.6, 12.6] | [11.3, 21.4] | [13.5, 17.7] | [10.1, 13.2] | [18.5, 19.3] |  |
|  | Q5 | 5.5 | 21.8 | 16.3 | 13.7 | 8.6 | 7.8 | 11.7 | 8.4 | 18.9 | 100 |
|  |  | [4.3, 7.1] | [21.3, 22.2] | [14.7, 18.0] | [12.4, 15.2] | [6.7, 11.1] | [6.1, 10.0] | [9.9, 13.7] | [7.1, 9.8] | [18.5, 19.2] |  |
| **Landed** | Yes | 73.6 | 11.5 | *** | 73.9 | 82.5 | 71.4 | 78.0 | 33.1 | 24.6 | 24.6 |
| **Immigrant** |  | [70.5, 76.5] | [11.1, 11.8] |  | [72.2, 75.7] | [79.3, 85.2] | [65.8, 76.5] | [75.3, 80.5] | [30.7, 35.7] | [24.1, 25.0] |  |
| **status** | No | 6.2 | 1.3 | *** | 8.1 | 5.2 | 7.6 | 5.0 | 2.8 | 2.4 | 27.0 |
|  |  | [4.7, 8.2] | [1.2, 1.5] |  | [7.1, 9.1] | [3.8, 6.9] | [5.5, 10.4] | [3.9, 6.3] | [2.1, 3.7] | [2.2 ,2.6] |  |
|  | Born in | 20.2 | 87.1 | 98.8 | 17.6 | 12.1 | 20.8 | 16.7 | 17.4 | 70.7 | 97.6 |
|  | Canada | [17.6, 23.0] | [86.8, 87.5] | [98.4, 99.2] | [16.1, 19.2] | [9.7, 15.1] | [15.9, 26.7] | [14.5, 19.3] | [15.7, 19.3] | [70.2, 71.1] |  |

**Notes**: Education: Low = less than secondary school graduation; Medium = secondary school graduation; High = certificate diploma or university degree. Categories Low, Medium, and High included 98.4% of study population; the remained 1.6% correspond to the category Not stated. For Landed immigrant status, the categories Yes, No, and Born in Canada included 97.6% of study population; the remained 2.4% correspond to the categories Don’t know, Refusal or Not stated.

*** = do not meet the confidentiality requirements to be published.

**Table S4**. Mammography screening. Women 50 to 74 years old. Observations = 29,779, Weighted population size = 2,733,152. Weighted frequencies expressed as percentages (%) with 95% CI in parentheses.

| **Question** | **Answer** | **%/CI/n** | **Black** | **Latin American** | **White** | **Indigenous** | **Southeast Asian** | **West Asian** | **South Asian** | **Other/Not declared** | **Total** |
| --- | --- | --- | --- | --- | --- | --- | --- | --- | --- | --- | --- |
| **Had a** | Yes | w% | 89.3 | *** | 91.1 | 88.4 | 80.5 | 86.9 | 80.8 | 81.5 | 89.7 |
| **Mammography?** |  | 95%CI | 83.2, 93.3 |  | 90.5, 91.7 | 85.6,  90.7 | 74.1,  85.6 | 78.7, 92.3 | 71.6, 87.5 | 74.1,  87.1 | 89.0, 90.4 |
|  |  | n | 383** | | 24,101 | 931 | 586 | 353** | | 676 | 27,030 |
|  | No+ | w% | 10.8 | *** | 8.9 | 11.6 | 19.5 | 13.1 | 19.2 | 18.6 | 10.3 |
|  |  | 95%CI | 6.7, 16.8 |  | 8.4, 9.5 | 9.3,  14.4 | 14.4,  25.9 | 7.7,  21.4 | 12.5, 28.4 | 12.9,  25.9 | 9.6,  11.0 |
|  |  | n | 47** | | 2269 | 148 | 116 | 67** | | 102 | 2,749 |
| **When was the** | ≤ 2yrs | w% | 78.5 | *** | 76.7 | 72.5 | 70.4 | 86.6 | 79.2 | 72.8 | 76.4 |
| **Last time?** |  | 95%CI | 70.3, 85 |  | 75.9, 77.6 | 66.7,  77.5 | 64.1,  76.1 | 78.0,  92.2 | 69.3, 86.5 | 67.2, 77.8 | 75.5, 77.2 |
|  |  | n | 296** | | 18,283 | 666 | 424 | 266** | | 469 | 20,404 |
|  | > 2yrs* | w% | 21.5 | *** | 23.3 | 27.5 | 29.6 | 13.4 | 20.8 | 27.2 | 23.6 |
|  |  | 95%CI | 15, 29.7 |  | 22.4, 24.1 | 22.5,  33.3 | 23.9,  36.0 | 7.8,  22.0 | 13.5, 30.7 | 22.2,  32.8 | 22.8, 24.5 |
|  |  | n | 87** | | 5,818 | 265 | 162 | 87** | | 207 | 6,626 |

**Notes:** No+ includes the categories of No, Don’t know, Refusal or Not stated.

> 2yrs* includes the categories of More than 2 years, Don’t know, Refusal or Not stated.

** These groups were combined to fulfill Statistics Canada requirements for publication.

*** = do not meet the confidentiality requirements to be published.

w%: weighted frequency.
